# Bots and Misinformation Spread on Social Media: Implications for COVID-19

**DOI:** 10.2196/26933

**Published:** 2021-05-20

**Authors:** McKenzie Himelein-Wachowiak, Salvatore Giorgi, Amanda Devoto, Muhammad Rahman, Lyle Ungar, H Andrew Schwartz, David H Epstein, Lorenzo Leggio, Brenda Curtis

**Affiliations:** 1 Intramural Research Program National Institute on Drug Abuse Baltimore, MD United States; 2 Department of Computer and Information Science University of Pennsylvania Philadelphia, PA United States; 3 Department of Computer Science Stony Brook Unversity Stony Brook, NY United States

**Keywords:** COVID-19, coronavirus, social media, bots, infodemiology, infoveillance, social listening, infodemic, spambots, misinformation, disinformation, fake news, online communities, Twitter, public health

## Abstract

As of March 2021, the SARS-CoV-2 virus has been responsible for over 115 million cases of COVID-19 worldwide, resulting in over 2.5 million deaths. As the virus spread exponentially, so did its media coverage, resulting in a proliferation of conflicting information on social media platformsa so-called infodemic. In this viewpoint, we survey past literature investigating the role of automated accounts, or bots, in spreading such misinformation, drawing connections to the COVID-19 pandemic. We also review strategies used by bots to spread (mis)information and examine the potential origins of bots. We conclude by conducting and presenting a secondary analysis of data sets of known bots in which we find that up to 66% of bots are discussing COVID-19. The proliferation of COVID-19 (mis)information by bots, coupled with human susceptibility to believing and sharing misinformation, may well impact the course of the pandemic.

## Introduction

Globally, 2020 has been characterized by COVID-19, the disease caused by the SARS-CoV-2 virus. As of March 2021, the COVID-19 pandemic has been responsible for over 115 million documented cases, resulting in over 2.5 million deaths. The United States accounts for 24.9% of the worlds COVID-19 cases, more than any other country [1].

As the virus spread across the United States, media coverage and information from online sources grew along with it [2]. Among Americans, 72% report using an online news source for COVID-19 information in the last week, with 47% reporting that the source was social media [3]. The number of research articles focusing on COVID-19 has also grown exponentially; more research articles about the disease were published in the first 4 months of the COVID-19 pandemic than throughout the entirety of the severe acute respiratory syndrome (SARS) and Middle East respiratory syndrome (MERS) pandemics combined [4]. Unfortunately, this breadth, and the speed with which information can travel, sets the stage for the rapid transmission of misinformation, conspiracy theories, and fake news about the pandemic [5]. One study found that 33% of people in the United States report having seen a lot or a great deal of false or misleading information about the virus on social media [3]. Dr Tedros Adhanom Ghebreyesus, the Director-General of the World Health Organization, referred to this accelerated flow of information about COVID-19, much of it inaccurate, as an infodemic [6].

Though the pandemic is ongoing, evidence is emerging regarding COVID-19 misinformation on social media. Rumors have spread about the origin of the virus, potential treatments or protections, and the severity and prevalence of the disease. In one sample of tweets related to COVID-19, 24.8% of tweets included misinformation and 17.4% included unverifiable information [7]. The authors found no difference in engagement patterns with misinformation and verified information, suggesting that myths about the virus reach as many people on Twitter as truths. A similar study demonstrated that fully false claims about the virus propagated more rapidly and were more frequently liked than partially false claims. Tweets containing false claims also had less tentative language than valid claims [8].

This trend of misinformation emerging during times of humanitarian crises and propagating via social media platforms is not new. Previous research has documented the spread of misinformation, rumors, and conspiracies on social media in the aftermath of the 2010 Haiti earthquake [9], the 2012 Sandy Hook Elementary School shooting [10], Hurricane Sandy in 2012 [11], the 2013 Boston Marathon bombings [12,13], and the 2013 Ebola outbreak [14].

Misinformation can be spread directly by humans, as well as by automated online accounts, colloquially called bots. Social bots, which pose as real (human) users on platforms such as Twitter, use behaviors like excessive posting, early and frequent retweeting of emerging news, and tagging or mentioning influential figures in the hope they will spread the content to their thousands of followers [15]. Bots have been found to disproportionately contribute to Twitter conversations on controversial political and public health matters, although there is less evidence they are biased toward one side of these issues [16-18].

This paper combines a scoping review with an unpublished secondary analysis, similar in style to Leggio et al [19] and Zhu et al [20]. We begin with a high-level survey of the current bot literature: how bots are defined, what technical features distinguish bots, and the detection of bots using machine learning methods. We also examine how bots spread information, including misinformation, and explore the potential consequences with respect to the COVID-19 pandemic. Finally, we analyze and present the extent to which known bots are publishing COVID-19related content.

## What Are Bots?

Before addressing issues surrounding the spread of misinformation, we provide a definition of bots, describe their typical features, and explain how detection algorithms identify bots.

### Definition and Identification

Bots, shorthand for software robots, come in a large variety of forms. Bots are typically automated in some fashion, either fully automated or human-in-the-loop. There is a common conception that all bots spam or spread malware, but this is not the case. Some bots are benign, like the Twitter account @big_ben_clock, which impersonates the real Big Ben clock by tweeting the time every hour [21]. Others have even been used for social good, such as Botivist, which is a Twitter bot platform used for recruiting volunteers and donations [22]. Groups of bots can function in coordination with each other, forming what are called botnets [23]. One botnet of roughly 13,000 bot accounts was observed tweeting about Brexit, with most of these bot accounts disappearing from Twitter shortly after the vote [24]. Bots of all types are ubiquitous on social media and have been studied on Reddit [25,26], Facebook [27], YouTube [28], and Twitter [29], among other platforms.

Given their large variety, bots are often organized into subclasses, a selection of which we discuss here. Content polluters are one subclass; these are accounts that disseminate malware and unsolicited content. Traditional spambots, another subclass, are designed to be recognizable as bots [30]. Social botsa newer, more advanced type of bot [31-33]use a computer algorithm that automatically produces content and interacts with humans on social media, trying to emulate and possibly alter their behavior. There are also hybrid human-bot accounts (often called cyborgs) [34], which exhibit human-like behavior and messages through loosely structured, generic, automated messages and from borrowed content copied from other sources [35]. It is not always clear which category a bot may fall into (eg, if a given social bot is also a cyborg).

Various methods have been used to identify bots in the wild, so as to build the data sets of known bots used to train bot-detection algorithms. One method, the social honeypot [36], mimics methods traditionally used by researchers to monitor hacker activity [37] and email harvesting [38]. Specifically, social honeypots are fake social media profiles set up with characteristics desirable to spammers, such as certain demographics, relationship statuses, and profile pictures [39]. When bots attempt to spam the honeypots (by linking malware-infested content or pushing product websites), researchers can easily identify them.

### Technical Features of Bots

#### Overview

Features that distinguish bots from humans roughly fall into three categories: (1) network properties, such as hashtags and friend/follower connections, (2) account activity and temporal patterns, and (3) profile and tweet content. These feature categories have the advantage of being applicable across different social media platforms [27].

#### Network Properties

Networks based on friend/follower connections, hashtag use, retweets, and mentions have been used in a number of studies that seek to identify social bots [40-43], exploiting network homophily (ie, humans tend to follow other humans and bots tend to follow other bots). As bots become more sophisticated, network properties become less indicative of them; studies have found groups of bots that were able to build social networks that mimic those of humans [44].

#### Account Activity and Temporal Patterns

Patterns of content generation can be good markers of bots. Bots compose fewer original tweets than humans, but retweet others tweets much more frequently, and have a shorter time interval between tweets [40]. Ferrara et al [31] found that humans are retweeted by others more than are bots, suggesting that bots may struggle to compose convincing or interesting tweets. However, many others have found this not to be the case [15,16,33]. Finally, humans typically modify their behavior during each online session; as the session progresses, the density of new tweets decreases. Bots do not engage in these sessions of social media usage, and accordingly do not modify their behavior [45].

#### Profile and Tweet Content

Profile metadata such as account age and username can be used to identify social bots. Ferrara et al [31] showed that bots have shorter account age (ie, the accounts were created more recently), as well as longer usernames. Automatic sentiment analysis of tweet content has also been studied as a means of distinguishing bots from humans. One study found humans expressed stronger positive sentiment than bots, and that humans more frequently flip-flopped in their sentiment [42].

### Detection of Bots

Over the past decade, several teams have sought to develop algorithms that successfully identify bots online. Social media platforms use similar algorithms internally to remove accounts likely to be bots. These algorithms originated in early attempts to identify spam emails [46], social phishing [47], and other types of cybercrimes [37]. With the advent of online communities, cybercriminals turned their attention to these sites, eventually creating fake, automated accounts at scale [48]. Table 1 provides a summary of several prominent papers on bot identification. We note that the details of specific machine learning algorithms are beyond the scope of this paper and therefore are not included in this manuscript.

**Table 1:** Review of state-of-the-art detection of bots on Facebook and Twitter.

Type and reference		Platform	Number of accounts	Features				Model		Metric		Predictive accuracy
				N^a^	T^b^	C^c^						
**Human judgment (manual annotation)**												
	Cresci et al (2017) [33]	Twitter	928				Manual annotation		F1-score		0.57	
**Automatic methods**												
	Ahmed and Abulaish (2013) [27]	Facebook and Twitter	320 (Facebook), 305 (Twitter)				Nave Bayes, decision trees, rule learners		Detection rate		0.96 (Facebook), 0.99 (Twitter)	
	Dickerson et al (2014) [42]	Twitter	897				Gradient boosting		Area under the curve		0.73	
	Cresci et al (2017) [33]	Twitter	928				Digital DNA sequences		F1-score		0.92	
	Varol et al (2017) [41]	Twitter	21,000				Random forests		Area under the curve		0.95	
	Kudugunta and Ferrara (2018) [49]	Twitter	8386				AdaBoost		Area under the curve		>0.99	
	Mazza et al (2019) [50]	Twitter	1000				Long short-term memory networks		F1-score		0.87	
	Santia et al (2019) [51]	Facebook	1000				Support vector machines, decision trees, Nave Bayes		F1-score		0.72	
	Yang et al (2020) [52]	Twitter	137,520				Random forests		Area under the curve		0.60-0.99	

^a^N: network properties.

^b^T: account activity and temporal patterns.

^c^C: profile and tweet content.

The first reference in Table 1 involved a manual annotation task in which raters were asked to label a Twitter account as human or bot. The fourth study listed in the table is the same as the first study; in this study, the same data set was evaluated by both human annotators and machine learning methods [33]. There was a large discrepancy in predictive accuracy (F1-score) between the two methods: 0.57 for the human annotators versus 0.92 for the automated method. Stated another way, human participants correctly identified social bots less than 25% of the time, though they were quite good at identifying genuine (human) accounts (92%) and traditional spambots (91%). These results suggest that social bots have a very different online presence from traditional spambots, or content pollutersand that this presence is convincingly human. Even if the human annotators are compared to the lowest scoring automated method (which we note is in a different domain and, thus, not directly comparable), the machine learning algorithm still provides a considerable boost in F1-score (0.57 versus 0.72).

There is no good way to compare all automated methods directly, as data sets are typically built in a single domain (ie, a single social media platform) and rapid advances in machine learning techniques prevent comparisons between models published even a few years apart. Furthermore, results suggest that models trained on highly curated bot data sets (eg, groups of accounts promoting certain hashtags or spamming a particular honeypot) may not perform well at detecting bots in other contexts. Yang et al [52] used a large number of publicly available bot data sets, training machine learning models on each set and testing them on those remaining. The result was a wide range of predictive accuracies across different bot data sets.

## How Do Bots Amplify and Spread Misinformation?

We adopt the definition of misinformation used by Treen and colleagues: misleading [or false] information that is created and spread, regardless of intent to deceive [53]. For the purposes of this paper, we include fake news and false conspiracy theories under this umbrella term.

Many features of bots likely enable them to be super-spreaders of misinformation. Bots have been shown to retweet articles within seconds of their first being posted, contributing to the articles going viral [15]. Moreover, the authors of this study found that 33% of the top sharers of content from low-credibility sources were likely to be bots, significantly higher than the proportion of bots among top sharers of fact-checked content. Similarly, in a study of bots and anti-vaxxer tweets, Yuan et al [18] found that bots were hyper-social, disproportionately contributing to content distribution. Bots also employ the strategy of mentioning influential users, such as @realDonaldTrump, in tweets linking to false or misleading articles, and are more likely to do so than their human counterparts [15]. The hope is that these users will share the article with their many followers, contributing to its spread and boosting its credibility. Verified (blue check) Twitter users, often celebrities, have been shown to both author and propagate COVID-19related misinformation [54]. Interestingly, the frequency of false claims about the 2020 election dropped dramatically in the week after former president Donald Trump was removed from the platform [55].

In light of findings that humans are largely unable to distinguish social bots from genuine (human) accounts [33], it is likely that humans unknowingly contribute to the spread of misinformation as well. Accordingly, one study found that in regard to low-credibility content, humans retweet bots and other humans at the same rate [15]. Similarly, Vosoughi et al [56] found that fake news articles spread faster on Twitter than true news articles because humans, not bots, were more likely to retweet fake articles. Given human susceptibility to both automated accounts and fake news, some have warned that intelligent social bots could be leveraged for mass deception or even political overthrow [57].

There is reason to believe that bots have already infiltrated political conversations online. Leading up to the 2016 presidential election in the United States, 20% of all political tweets originated from accounts that were likely to be bots [16]. While it did not specifically implicate bots, one study found that a majority of fake or extremely biased news articles relating to the 2016 election were shared by unverified accountsthat is, accounts that were not confirmed to be human [58]. There is also evidence that bots spread misinformation in the 2017 French presidential election, though ultimately the bot campaign was unsuccessful, in part because the human users who engaged with the bots were mostly foreigners with no say in the election outcome [59]. Bot strategies specifically relevant to political campaigns include hashtag hijacking, in which bots adopt an opponents hashtags in order to spam or otherwise undermine them, as well as flagging their opponents legitimate content in the hopes it gets removed from the platform [60].

## Where Do Bots Come From?

The origin of social bots is a challenging question to answer. Given the aforementioned concerns of political disruption by social bots, one may assume that foreign actors create social bots to interfere with political processes. Indeed, the Mueller report found evidence of Russian interference in the 2016 US election via social media platforms [61], and Twitter reports removing over 50,000 automated accounts with connections to Russia [62]. However, locating the origin of a social media account is difficult, as tweets from these accounts are rarely geotagged. Rheault and Musulan [63] proposed a methodology to identify clusters of foreign bots used during the 2019 Canadian election using uniform manifold approximation and projection combined with user-level document embeddings. Simply put, the authors constructed communities of users via linguistic similarities, and identified members significantly outside these communities as foreign bots.

Of note, studies have shown that a majority of social bots focusing on election-related content originate domestically [63]. Reasons for a candidate or their supporters to employ social bots may be relatively benign, such as boosting follower counts or sharing news stories, or they may involve smear campaigns [64].

While the ability to investigate the origin and motive of social bots is difficult, the means to create a social bot are fairly easy to access. Social bots are available for purchase on the dark web, and there are tens of thousands of codes for building social bots on free repositories like GitHub [65]. Of note, the top contributors of bot-development tools for mainstream social media sites are the United States, the United Kingdom, and Japan. The authors of this paper also note the intelligence and capabilities of these freely available bots may be overstated.

## Are Bots Tweeting About COVID-19?

In light of the COVID-19 infodemic and findings that social bots have contributed to misinformation spread in critical times, we sought to assess the number of known Twitter bots producing COVID-19related content. To this end, we gathered a number of publicly available bot data sets from the Bot Repository [66]. These data sets include both traditional spambots and social bots that were first identified through a number of different methods (see the original papers for more details).

Using the open-source Python package TwitterMySQL [67], which interfaces with the Twitter application programming interface (API), we were able to pull all tweets from 2020 for each bot in the combined data set. Of note, Twitters API limits access to tweets and account information available at the time of collection. Tweets and accounts that have been deleted or made private since originally appearing in one of the above papers are not made available, meaning we had less data than what was reported in the original papers. Our final data set consisted of 3.03 million tweets from 3953 bots, with an average of 768.9 (SD 1145.4) tweets per bot, spanning January 1, 2020, to August 21, 2020.

From these data, we pulled tweets using a set of 15 COVID-19related keywords, which have previously been used to identify COVID-19 tweets in a study tracking mental health and psychiatric symptoms over time [68]. Sample keywords include #coronavirus, #covid19, and #socialdistancing. We then counted the number of accounts that mentioned these keywords in tweets since January 2020. Table 2 shows the percentage of bot accounts in each data set currently tweeting about COVID-19. Original sample size refers to the number of bots identified in this data set, while current sample size is the number of currently active bots (ie, tweeting in 2020). Between 53% (96/182) and 66% (515/780) of these bots are actively tweeting about COVID-19.

**Table 2:** Open-source data sets of bots discussing COVID-19^a^.

Reference	Year	Original sample size, n	Current sample size, n	Bots discussing COVID-19, n (%)
Lee et al [36]	2011	22,223	2623	1427 (54)
Varol et al [41]	2017	826	292	164 (56)
Gilani et al [69]	2017	1130	780	515 (66)
Cresci et al [33]	2017	4912	77	48 (62)
Mazza et al [50]	2019	391	182	96 (53)

^a^Original sample size is the number of bot IDs publicly released on the Bot Repository, while current sample size is the number of active accounts tweeting in 2020. Percentage discussing COVID-19 is the percentage of bots with at least one tweet containing a COVID-19 keyword out of those active in 2020.

## Implications for the COVID-19 Pandemic

Here we have shown that a majority of known bots are tweeting about COVID-19, a finding that corroborates similar studies [68,70]. Early in the pandemic, one study found that 45% of COVID-19related tweets originate from bots [71], although Twitter has pushed back on this claim, citing false-positive detection algorithms [72]. Another study showed that COVID-19 misinformation on Twitter was more likely to come from unverified accountsthat is, accounts not confirmed to be human [7]. In an analysis of 43 million COVID-19related tweets, bots were found to be pushing a number of conspiracy theories, such as QAnon, in addition to retweeting links from partisan news sites [73]. Headlines from these links often suggested that the virus was made in Wuhan laboratories or was a biological weapon.

One limitation of our study is that we did not investigate the validity of COVID-19related claims endorsed by bots in our analyses. It may be that bots are largely retweeting mainstream news sources, as was the case in a recent study of bots using #COVID19 or #COVID-19 hashtags [68]. However, previous research has connected bots to the spread of misinformation in other public health domains, such as vaccines [30] and e-cigarettes [74], and unsubstantiated medical claims surrounding the use of marijuana [75].

Such misinformation can have detrimental consequences for the course of the COVID-19 pandemic. Examples of these real-world consequences include shortages of hydroxychloroquine (a drug that is crucial for treating lupus and malaria) due to increased demand from people who believe it will protect them from COVID-19 [76,77]. This drug has been promoted as a preventative against COVID-19 on social media, even though several randomized controlled trials have found it ineffective, [78,79], and the National Institutes of Health recently halted its own trial due to lack of effectiveness [80]. Moreover, belief in conspiracy theories about COVID-19 is associated with a decreased likelihood of engaging in protective measures such as frequent handwashing and social distancing, suggesting that misinformation may even contribute to the severity of the pandemic [81]. In addition, exposure to misinformation has been negatively correlated with intention to take a COVID-19 vaccine [82].

We are certainly not the first to express concern with viral misinformation; in May 2020, Twitter began labeling fake or misleading news related to COVID-19 in an effort to ensure the integrity of information shared on the platform [83]. Facebook introduced even more controls, such as organizing the most vetted articles at the top of the news feed, banning antimask groups, and sending antimisinformation messages to users who have shared fake news [84]. However, these measures are designed to target humans. In light of the numerous viral rumors relating to COVID-19 and the US response to the pandemic, we believe that bots likely contributed to their spread.

Major social media platforms like Twitter and Facebook do have methods to curtail suspected bots. In 2018, Twitter banned close to 70 million suspicious accounts in a matter of months [85]. Facebook banned 1.3 billion suspicious accounts in the third quarter of 2020. The platform estimates these accounts represent 5% of its worldwide monthly active users. The vast majority of suspicious accounts were identified using automated detection methods, but 0.7% were first flagged by human users, suggesting that everyday Facebook users concerned about malicious activity on the platform can contribute to efforts to ban these accounts [86].

Mitigation of the harmful effects of social bots can also occur at the policy level. In 2018, California became the first and only state to pass a law requiring social bots to identify themselves as such [87]. In 2019, Senator Dianne Feinstein proposed a similar bill federally; the bill would allow the Federal Trade Commission to enforce bot transparency and would prohibit political candidates from incorporating social bots in their campaign strategy [88]. The United States Congress has brought top executives from Facebook, Twitter, and Google to testify before Congress about Russian influence on their platforms in advance of the 2016 election [89]. Scholars have interpreted these actions as a sign that the government wishes to maintain the right to regulate content on social mediaa prospect that brings concerns of its own [90]. Presently, content problems on social media platforms are almost exclusively dealt with by the owners of those platforms, usually in response to user complaints, but in the coming years we may see an increase in government oversight on these platforms, fueling concerns about state-sponsored censorship [91,92]. More fundamentally, some have argued that, before any actionable policy or automatic interventions can be enabled, ambiguities in both bot definitions and jurisdiction and authority need to be addressed [90].

Even as citizens, social media platforms, and policy makers converge on the notion that bots and misinformation are urgent problems, the methods used to address the issue have had mixed results. When social media platforms crack down on bots and misinformation, either through automated techniques or manual content moderation, they run the risk of censoring online speech and further disenfranchising minority populations. Content promotion and moderation can lead to arbitrary policy decisions that may be inconsistent across or even within platforms [93]. In one example, Facebook ignited a controversy when their moderators flagged a breastfeeding photo as obscene, leading to a large number of protests on both sides of the debate [94]. Automated methods suffer from similar drawbacks, with multiple studies showing that biases in machine learning models can have unintended downstream consequences [95]. For example, algorithms designed to detect hate speech were more likely to label a post as toxic when it showed markers of African American English [96].

Finally, there is a continued arms race between bot-detection algorithms and bot creators [21,33]. As bots inevitably become more intelligent and convincingly human, the means for identifying them will have to become more precise. We observed that the majority of known bots in a sample of publicly available data sets are now tweeting about COVID-19. These bots, identified between 2011 and 2019, were discovered before the pandemic and were originally designed for nonCOVID-19 purposes: promoting product hashtags, retweeting political candidates, and spreading links to malicious content. The COVID-19 pandemic will eventually end, but we have reason to believe social bots, perhaps even the same accounts, will latch on to future global issues. Additionally, we can expect bot generation techniques to advance, especially as deep learning methods continue to improve on tasks such as text or image generation [97,98]. Bot creators will continue to deploy such techniques, possibly fooling detection algorithms and humans alike. In the end, we should not expect current detection techniques, self-policing of social media platforms, or public officials alone to fully recognize, or adequately address, the current landscape of bots and misinformation.

## Abbreviations

API: application programming interface
